# Appreciation message

**DOI:** 10.1590/S2237-96222024V33E2023030.EN

**Published:** 2024-03-11

The Editorial Core Group of Epidemiology and Health Services: Journal of the Brazilian National Health System (*Epidemiologia e Serviços de Saúde: revista do SUS* - RESS) extends its thanks to the *ad hoc* reviewers listed below for their collaboration and commitment to the careful assessment of manuscripts in 2023, which greatly contributed to the quality, independence and ethical rigor of the journal.

**Table:** 

Name	Institution	City	State	Country
Adalgisa Peixoto Ribeiro	Universidade Federal de Minas Gerais	Belo Horizonte	MG	Brazil
Adriana Ilha da Silva	Universidade Federal do Espírito Santo	Vitória	ES	Brazil
Adriana Skamvetsakis	Centro Regional de Referência em Saúde do Trabalhador da Região dos Vales	Santa Cruz do Sul	RS	Brazil
Aichely Rodrigues da Silva	Universidade Estadual da Região Tocantina do Maranhão	Imperatriz	MA	Brazil
Airton José Rombaldi	Universidade Federal de Pelotas	Pelotas	RS	Brazil
Aline Macarevich Condessa	Prefeitura Municipal de Porto Alegre	Porto Alegre	RS	Brazil
Alisson Diego Machado	Universidade de São Paulo	São Paulo	SP	Brazil
Aluísio de Oliveira	Instituto Federal de Educação, Ciência e Tecnologia do Sudeste de Minas Gerais	Juiz de Fora	MG	Brazil
Ana Carolina Micheletti Gomide Nogueira de Sá	Universidade Federal de Minas Gerais	Belo Horizonte	MG	Brazil
Ana Cristina Gonçalves Vaz dos Reis	Fundação Oswaldo Cruz	Rio de Janeiro	RJ	Brazil
Ana Débora Assis Moura	Secretaria da Saúde do Estado do Ceará	Fortaleza	CE	Brazil
Ana Lúcia Sartori	Universidade Federal de Mato Grosso	Sinop	MT	Brazil
Ana Luiza Gomes Domingos	Universidade de São Paulo	São Paulo	SP	Brazil
Ana Paula Gomes dos Santos	Secretaria de Saúde do Estado do Rio Grande do Sul	Porto Alegre	RS	Brazil
Ana Sílvia Scavacini Marinonio	Universidade Federal de São Paulo	São Paulo	SP	Brazil
André Luiz Lima	Prefeitura de Natal	Natal	RN	Brazil
Andréa Cronemberger Rufino	Universidade Federal do Piauí	Teresina	PI	Brazil
Camilo Molino Guidoni	Universidade Estadual de Londrina	Londrina	PR	Brazil
Carolina Paes Torres Mantovani	Universidade de São Paulo	São Paulo	SP	Brazil
Carolina Maia Martins Sales	Universidade Federal do Espírito Santo	Vitória	ES	Brazil
Caroline Mary Gurgel Dias Florencio	Universidade Federal do Ceará	Fortaleza	CE	Brazil
Cássia Cristina Pinto Mendicino	Secretaria Municipal de Saúde de Contagem/Diretoria de Vigilância Epidemiológica	Contagem	MG	Brazil
Cassio Hartmann	Instituto Federal de Alagoas	Maceió	AL	Brazil
Cesar Luiz Silva Junior	Escola Nacional de Saúde Pública Sergio Arouca	Rio de Janeiro	RJ	Brazil
Claudio Maierovitch Pessanha Henriques	Fundação Oswaldo Cruz	Brasília	DF	Brazil
Cleber Cremonese	Universidade Federal da Bahia	Salvador	BA	Brazil
Conceição Maria Oliveira	Secretaria de Saúde do Recife	Recife	PE	Brazil
Cristiane Lara Mendes-Chiloff	Faculdade de Medicina de Botucatu	Botucatu	SP	Brazil
Dalila Machado Botelho Oliveira	Organização Pan-Americana da Saúde/Organização Mundial da Saúde	Brasília	DF	Brazil
Danelle da Silva Nascimento	Hospital Universitário Júlio Maria Bandeira de Mello	Cajazeiras	PB	Brazil
Daniela de Almeida Pereira Duarte	Universidade Federal de Viçosa	Viçosa	MG	Brazil
Daniele Maria Pelissari	Ministério da Saúde	Brasília	DF	Brazil
Danielle Andreza da Cruz Ferreira	Universidade Federal de Minas Gerais	Belo Horizonte	MG	Brazil
Danillo Gonçalves de Barros	Escola Nacional de Saúde Pública Sergio Arouca	Rio de Janeiro	RJ	Brazil
Danúbia Hillesheim	Universidade Federal de Santa Catarina	Florianópolis	SC	Brazil
Deise Campos Cardoso	Fundação Hospitalar do Estado de Minas Gerais	Belo Horizonte	MG	Brazil
Denise Eliziana de Souza	Escola Nacional de Saúde Pública Sergio Arouca	Rio de Janeiro	RJ	Brazil
Denise Lopes Porto	Ministério da Saúde	Brasília	DF	Brazil
Dinar Duarte Vasconcelos	Universidade do Estado do Pará	Belém	PA	Brazil
Elaine Cristina Navarro	Faculdade Eduvale de Avaré	Avaré	SP	Brazil
Eleny Guimarães-Teixeira	Fundação Souza Marques	Rio de Janeiro	RJ	Brazil
Eliane Rolim Holanda	Universidade Federal da Paraíba	João Pessoa	PB	Brazil
Eliete Albano de Azevedo Guimarães	Universidade Federal de São João del-Rei	Divinópolis	MG	Brazil
Elizabeth Angélica Salinas Redolledo	Universidade de São Paulo	São Paulo	SP	Brazil
Elizabeth do Nascimento	Universidade Federal de Pernambuco	Recife	PE	Brazil
Eunaihara Marques	Instituto de Assistência Médica ao Servidor Público Estadual	São Paulo	SP	Brazil
Fabiana Schuelter-Trevisol	Universidade do Sul de Santa Catarina	Tubarão	SC	Brazil
Fernanda Vieira Almeida	Universidade Federal de Pelotas	Pelotas	RS	Brazil
Fernando Silva Guimarães	Universidade Federal de Pelotas	Pelotas	RS	Brazil
Flavia Cristina Drumond Andrade	University of Illinois at Urbana Champaign	-	-	united States
Flávio Fernando Batista Moutinho	Universidade Federal Fluminense	Niterói	RJ	Brazil
Franciele Silvia Carlo	Universidade Federal de Mato Grosso	Cuiabá	MT	Brazil
Frederico Alves Dias	Secretaria de Saúde do Estado do Paraná	Curitiba	PR	Brazil
Gabriela Callo Quinte	Universidade Federal do Espírito Santo	Vitória	ES	Brazil
Gabriela Avila Marques	Universidade Federal de Pelotas	Pelotas	RS	Brazil
Galba Freire Moita	Fundação Oswaldo Cruz	Fortaleza	CE	Brazil
Glaucia Maria Bressan	Universidade Tecnológica Federal do Paraná	Cornélio Procópio	PR	Brazil
Gustavo Libério de Paulo	Pontifícia Universidade Católica de Minas Gerais	Contagem	MG	Brazil
Gustavo Magno Baldin Tiguman	Universidade Estadual de Campinas	Campinas	SP	Brazil
Hayla Nunes da Conceição	Universidade Federal do Piauí	Teresina	PI	Brazil
Heloísa do Nascimento de Moura Meneses	Universidade Federal do Oeste do Pará	Santarém	PA	Brazil
Hernane Guimarães dos Santos Junior	Universidade Federal do Oeste do Pará	Santarém	PA	Brazil
Indianara Maria Barros Canuto	Secretaria de Saúde do Recife	Recife	PE	Brazil
Ione Ayala Gualandi de Oliveira	Fundação Oswaldo Cruz	Rio de Janeiro	RJ	Brazil
Ione Jayce Ceola Schneider	Novartis Biociências	-	-	Switzerland
Italo Wesley Oliveira Aguiar	Universidade Federal do Ceará	Fortaleza	CE	Brazil
Itamar Bento Claro	Instituto Nacional de Câncer	Rio de Janeiro	RJ	Brazil
Jaquelline Monte Stevanato	FIMCA Vilhena	Vilhena	RO	Brazil
Jardeliny Corrêa da Penha	Universidade Federal do Piauí	Floriano	PI	Brazil
Javier Sánchez Doncell	Universidad de Buenos Aires	-	-	Argentina
Jefferson Paixão Cardoso	Universidade Estadual do Sudeste da Bahia	Candeias	BA	Brazil
Jesem Douglas Yamall Orellana	Fundação Oswaldo Cruz/Instituto Leônidas e Maria Deane	Manaus	AM	Brazil
José Anael Neves	Universidade Estadual do Ceará	Fortaleza	CE	Brazil
José Carlos da Silva Melo	Universidade Federal da Paraíba	João Pessoa	PB	Brazil
José Romério Rabêlo Melo	Agência Nacional de Vigilância Sanitária	Brasília	DF	Brazil
Josivania Arrais de Figueiredo	Ministério da Saúde	Brasília	DF	Brazil
Juliana Gonçalves Reis	Fundação Oswaldo Cruz	Rio de Janeiro	RJ	Brazil
Juraci Almeida Cesar	Universidade Federal do Rio Grande	Porto Alegre	RS	Brazil
Kamila Tiemann Gabe	Universidade de São Paulo	São Paulo	SP	Brazil
Kátia Jakovljevic Pudla Wagner	Universidade Federal de Santa Catarina	Curitibanos	SC	Brazil
Katiane Pereira Braga	Instituto Federal de Educação, Ciência e Tecnologia do Tocantins	Araguaína	TO	Brazil
Kellyn Kessiene de Sousa Cavalcante	Universidade Federal do Ceará	Fortaleza	CE	Brazil
Kesia Tomasi da Rocha	Universidade Católica do Rio Grande do Sul	Porto Alegre	RS	Brazil
Klauss Kleydmann Sabino Garcia	Ministério da Saúde	Brasília	DF	Brazil
Kleber dos Santos	Universidade Federal Fluminense	Niterói	RJ	Brazil
Lacita Menezes Skalinski	Universidade Estadual de Santa Cruz	Ilhéus	BA	Brazil
Lara Onofre Ferriani	Centro Universitário São Camilo	São Paulo	SP	Brazil
Laura Moreira Goularte	Universidade Federal de Pelotas	Pelotas	RS	Brazil
Léa de Freitas Amaral	Escola Nacional de Saúde Pública	Rio de Janeiro	RJ	Brazil
Leilane Morais Lopes	Universidade Federal do Rio de Janeiro	Rio de Janeiro	RJ	Brazil
Lélia Mendes Sobrinho de Oliveira	Universidade Federal da Bahia	Salvador	BA	Brazil
Lia Borges de Mattos Custodio	Universidade Estadual Paulista	Araçatuba	SP	Brazil
Lia Zumblick Machado	Universidade do Sul de Santa Catarina	Tubarão	SC	Brazil
Lídia Bezerra Barbosa	Universidade Federal de Alagoas	Maceió	AL	Brazil
Luan Nascimento da Silva	Universidade Federal de Pelotas	Pelotas	RS	Brazil
Lucia Helena Linheira Bisetto	Universidade Federal de Minas Gerais	Belo Horizonte	MG	Brazil
Luciana Rodrigues Perrone	Universidade Federal de Pelotas	Pelotas	RS	Brazil
Luísa Silveira da Silva	Universidade Federal de Pelotas	Pelotas	RS	Brazil
Mábia Milhomem Bastos	Universidade do Estado do Rio de Janeiro	Rio de Janeiro	RJ	Brazil
Marcela Lopes Bhering da Silva	Centro de Referência Professor Hélio Fraga	Rio de Janeiro	RJ	Brazil
Marcela Lopes Santos	Ministério da Saúde	Brasília	DF	Brazil
Márcio Denis Medeiros Mascarenhas	Universidade Federal do Piauí	Teresina	PI	Brazil
Marco Antônio Natal Vigilato	Organização Pan-Americana da Saúde/Organização Mundial da Saúde	Brasília	DF	Brazil
Marcos Aurélio Matos Lemões	Universidade Federal de Pelotas	Pelotas	RS	Brazil
Marcos Cláudio Signorelli	Universidade Federal do Paraná	Curitiba	PR	Brazil
Marcos Venicius Malveira de Lima	Secretaria de Estado de Saúde do Acre	Rio Branco	AC	Brazil
Maria Carmelita Maia	Instituto de Medicina Legal de Pernambuco	Recife	PE	Brazil
Maria Cristina Antunes Willemann	Conselho de Secretarias Municipais de Saúde de Santa Catarina	Florianópolis	SC	Brazil
Maria das Dores Nunes	Universidade de Brasília	Brasília	DF	Brazil
Maria Elizete de Almeida Araujo	Universidade Federal de Pelotas	Pelotas	RS	Brazil
Maria Isabel do Nascimento	Universidade Federal Fluminense	Niterói	RJ	Brazil
Maria Rita Bertolozzi	Universidade de São Paulo	São Paulo	SP	Brazil
Maria Rita Marques de Oliveira	Universidade Estadual Paulista “Júlio de Mesquita Filho”	São Paulo	SP	Brazil
Maria Rosana Issberner Panachão	Instituto Adolfo Lutz	São Paulo	SP	Brazil
Mariana Mapelli de Paiva	Instituto Federal do Norte de Minas Gerais	Belo Horizonte	MG	Brazil
Mario Jorge Sobreira da Silva	Instituto Nacional de Câncer	Rio de Janeiro	RJ	Brazil
Mário Ribeiro Alves	Universidade do Estado do Rio de Janeiro	Rio de Janeiro	RJ	Brazil
Maristela Goldnadel Monteiro	Universidade Federal de São Paulo	São Paulo	SP	Brazil
Matheus Henrique Pimenta-Zanon	Universidade Tecnológica Federal do Paraná	Curitiba	PR	Brazil
Maurício Polidoro	Ministério dos Povos Indígenas	Brasília	DF	Brazil
Mayara Secco Torres da Silva	Fundação Oswaldo Cruz	Rio de Janeiro	RJ	Brazil
Micheline da Silveira Mendes	Fundação Oswaldo Cruz	Recife	PE	Brazil
Nathalie Mendes Estima	Ministério da Saúde	Brasília	DF	Brazil
Nayore Tamie Takamiya	Universidade Federal de São Carlos	São Carlos	SP	Brazil
Neilane Bertoni	Instituto Nacional de Câncer	Rio de Janeiro	RJ	Brazil
Nicole Freitas de Mello	Ministério da Saúde	Brasília	DF	Brazil
Norma Suely de Oliveira Farias	Secretaria de Estado da Saúde de São Paulo	São Paulo	SP	Brazil
Orlando Luiz do Amaral Júnior	Universidade Federal de Santa Maria	Santa Maria	RS	Brazil
Osmar de Oliveira Cardoso	Universidade Federal do Piauí	Teresina	PI	Brazil
Pamila Cristina Lima Siviero	Universidade Federal de São Paulo	São Paulo	SP	Brazil
Patrícia Pinheiro de Freitas	Universidade Federal de Minas Gerais	Belo Horizonte	MG	Brazil
Paula Araujo Opromolla	Instituto Adolfo Lutz	São Paulo	SP	Brazil
Paulo Henrique Faria Domingues	Centro Universitário IMEPAC	Araguari	MG	Brazil
Paulo Ricardo Rocha Nogueira	Universidade Federal do Rio Grande do Sul	Porto Alegre	RS	Brazil
Pedro de Alcântara Brito Júnior	Ministério da Saúde	Brasília	DF	Brazil
Policardo Gonçalves da Silva	Universidade do Estado de Minas Gerais	Belo Horizonte	MG	Brazil
Pollyanna Micali	Centro Universitário Central Paulista	São Paulo	SP	Brazil
Priscila Stacul	ABEU Centro Universitário	Belford Roxo	RJ	Brazil
Rafaela Albuquerque	Universidade de Brasília	Brasília	DF	Brazil
Rafaela de Almeida Schiavo	Universidade Estadual Paulista “Júlio de Mesquita Filho”	São Paulo	SP	Brazil
Raquel Zanatta Coutinho	Universidade Federal de Minas Gerais	Belo Horizonte	MG	Brazil
Ricardo Franklin de Freitas Mussi	Universidade do Estado da Bahia	Salvador	BA	Brazil
Ricardo José de Paula e Guimarães	Instituto Evandro Chagas	Ananindeua	PA	Brazil
Riceli Rodeghiero Oliveira	Universidade Federal de Pelotas	Pelotas	RS	Brazil
Rivaldo Faria	Universidade Federal de Santa Maria	Santa Maria	RS	Brazil
Roger Keller Celeste	Universidade Federal do Rio Grande do Sul	Porto Alegre	RS	Brazil
Ronaldo Corrêa Ferreira da Silva	Instituto Nacional de Câncer	Rio de Janeiro	RJ	Brazil
Rosália Garcia Neves	Secretaria de Saúde do Rio Grande do Sul	Porto Alegre	RS	Brazil
Roudom Ferreira Moura	Secretaria de Estado da Saúde de São Paulo	São Paulo	SP	Brazil
Samilly Silva Miranda	Universidade Estadual de Feira de Santana	Feira de Santana	BA	Brazil
Sandra Mara Silva Brignol	Universidade Federal Fluminense	Niterói	RJ	Brazil
Sandra Costa Fonseca	Universidade Federal Fluminense	Niterói	RJ	Brazil
Sandra Garrido de Barros	Universidade Federal da Bahia	Salvador	BA	Brazil
Sarah Arangurem Karam	Universidade Federal de Pelotas	Pelotas	RS	Brazil
Sidney Marcel Domingues Marcel	Universidade de São Paulo	Ribeirão Preto	SP	Brazil
Silvana Márcia Bruschi Kelles	Pontifícia Universidade Católica de Minas Gerais	Belo Horizonte	MG	Brazil
Silvia Giselle Ibarra Ozcariz	Universidade Federal de Santa Catarina	Florianópolis	SC	Brazil
Silvio Barberato-Filho	Universidade de Sorocaba	Sorocaba	SP	Brazil
Suzana Madeira Diorio	Instituto Lauro de Souza Lima	Bauru	SP	Brazil
Tamara Leite Cortez	Prefeitura Municipal de São Paulo	São Paulo	SP	Brazil
Tatiana Rodrigues de Araujo Eleuterio	Universidade do Estado do Rio de Janeiro	Rio de Janeiro	RJ	Brazil
Tatiana Mingote Ferreira de Ázara	Instituto René Rachou	Belo Horizonte	MG	Brazil
Tayanny Margarida Menezes Almeida Biase	Universidade Estadual de Campinas	Campinas	SP	Brazil
Teresa Cristina Vieira Segatto	Secretaria de Saúde do Distrito Federal	Brasília	DF	Brazil
Thais Gularte Della Vechia	Universidade Federal de Pelotas	Pelotas	RS	Brazil
Thanise Sabrina Souza Santos	Universidade de São Paulo	São Paulo	SP	Brazil
Thiago Perez Moraes	Universidade Federal de Pelotas	Pelotas	RS	Brazil
Tiago Oliveira de Souza	Universidade Federal do Rio de Janeiro	Rio de Janeiro	RJ	Brazil
Vanessa Gomes	Universidade Federal do Amazonas	Manaus	AM	Brazil
Vanessa Moreira da Silva Soeiro	Universidade Federal do Maranhão	São Luís	MA	Brazil
Virginia Kagure Wachira	Ministério da Saúde	Brasília	DF	Brazil
Vivian Siqueira Santos Gonçalves	Universidade de Brasília	Brasília	DF	Brazil
Waneska Alexandra Alves	Universidade Federal de Juiz de Fora	Governador Valadares	MG	Brazil
Werner de Andrade Müller	Universidade Federal de Pelotas	Pelotas	RS	Brazil
Yluska Myrna Meneses Brandão e Mendes	Ministério da Saúde	Brasília	DF	Brazil

